# Word reading and translation in bilinguals: the impact of formal and informal translation expertise

**DOI:** 10.3389/fpsyg.2014.01302

**Published:** 2014-11-12

**Authors:** Adolfo M. García, Agustín Ibáñez, David Huepe, Alexander L. Houck, Maëva Michon, Carlos G. Lezama, Sumeer Chadha, Álvaro Rivera-Rei

**Affiliations:** ^1^National Scientific and Technical Research CouncilBuenos Aires, Argentina; ^2^School of Languages, National University of CórdobaCórdoba, Argentina; ^3^Laboratory of Experimental Psychology and Neuroscience, Institute of Cognitive Neurology, Favaloro UniversityBuenos Aires, Argentina; ^4^Laboratory of Cognitive and Social Neuroscience, Institute of Cognitive Neurology, Foundation Core on Neuroscience, Diego Portales UniversitySantiago, Chile; ^5^Universidad Autónoma del CaribeBarranquilla, Colombia; ^6^Centre of Excellence in Cognition and its Disorders, Australian Research CouncilSydney, NSW, Australia; ^7^University of TennesseeKnoxville, TN, USA

**Keywords:** word reading, word translation, concreteness effect, cognate effect, translation expertise, L2 proficiency

## Abstract

Studies on bilingual word reading and translation have examined the effects of lexical variables (e.g., concreteness, cognate status) by comparing groups of non-translators with varying levels of L2 proficiency. However, little attention has been paid to another relevant factor: translation expertise (TI). To explore this issue, we administered word reading and translation tasks to two groups of non-translators possessing different levels of informal TI (Experiment 1), and to three groups of bilinguals possessing different levels of translation training (Experiment 2). Reaction-time recordings showed that in all groups reading was faster than translation and unaffected by concreteness and cognate effects. Conversely, in both experiments, all groups translated concrete and cognate words faster than abstract and non-cognate words, respectively. Notably, an advantage of backward over forward translation was observed only for low-proficiency non-translators (in Experiment 1). Also, in Experiment 2, the modifications induced by translation expertise were more marked in the early than in the late stages of training and practice. The results suggest that TI contributes to modulating inter-equivalent connections in bilingual memory.

## INTRODUCTION

In psycholinguistics and cognitive psychology, translation and its subskills have become the object of inquiry of two sets of studies: (i) experiments on bilingual memory organization, using paradigms such as word reading and word translation with non-translators (Kroll and Stewart, 1994; for reviews, see French and Jacquet, 2004; Brysbaert and Duyck, 2010; Kroll et al., 2010); and (ii) investigations on the impact of translation expertise on both linguistic and non-linguistic functions (Fabbro et al., 1991; Bajo et al., 2000; Ibáñez et al., 2010; Yudes et al., 2012). Here, we pursue an intersection of both trends. Our goal is to explore how different types and levels of translation expertise impact on lexical access, both within and across languages.

Reading and translating words are part of everyday linguistic processing for bilinguals. Previous studies have shown that word reading is faster than word translation (Kroll and Stewart, 1994; La Heij et al., 1996). Also, in non-translators, lexical processes are usually faster in the native language (L1) than in the non-native language (L2), although such a difference is attenuated as L2 proficiency increases (Kroll and Stewart, 1994; Sholl et al., 1995; French and Jacquet, 2004).

As regards word translation, some experiments with non-translators have demonstrated directionality effects, typically with faster reaction times (RTs) in backward translation (BT, from L2 into L1) than forward translation (FT, from L1 into L2; Kroll and Stewart, 1994; Sholl et al., 1995; Choi, 2005; Bowers and Kennison, 2011). However, several studies have also reported the opposite effect as well as null results (de Groot et al., 1994; La Heij et al., 1996; de Groot and Poot, 1997; van Hell and de Groot, 1998a; Christoffels et al., 2003; Duyck and Brysbaert, 2008). Further evidence for directionality asymmetries comes from translation priming studies, with robust effects in the L1–L2 direction but less consistent effects in the L2–L1 direction (Kiran and Lebel, 2007).

Word translation is also sensitive to the stimuli’s concreteness, a semantic variable indicating activation of concept-level links. In a study on FT, de Groot (1992, Experiment 3) noted that concrete nouns were translated faster than abstract nouns, and that such an effect was larger for high- than for low-frequency words. For their part, de Groot et al. (1994) found a similar –though less statistically significant– directionality effect, but on the abstract as opposed to the concrete words. An asymmetric concreteness effect was also reported by van Hell and de Groot (1998a), but this time it was slightly larger for FT than BT. While not entirely consistent, the results show that concreteness modulates the directionality effect.

Another important variable is cognate status –i.e., the level of orthographic/phonological similarity between translation equivalents. Several studies have shown that cognates are processed faster than non-cognates (Costa et al., 2000), indicating stronger connections for words sharing sublexical properties across languages. Moreover, this effect is proportional to the degree of orthographic overlap between equivalents. Using a lexical decision task in L2, Dijkstra et al. (2010) found that RTs decreased as formal similarity increased across counterparts (e.g., *lamp-lamp*<*flood-vloed*<*song-lied*). Cognate facilitation was significant even for word pairs with only partial formal overlap (e.g., *guide-gids*, *rhythm-ritme*). As regards word translation, de Groot (1992, 1993) conducted an FT task and found that cognates were translated faster than non-cognates. More recently, Christoffels et al. (2003) assessed this effect across translation directions using cognate and non-cognate stimuli matched for frequency, length, and concreteness. Crucially, they found hat cognates were translated faster than non-cognates in both directions. Similarly, an advantage of cognates over non-cognates has been reported in other interlinguistic tasks, such as translation priming (de Groot and Nas, 1991) and cross-language word association (van Hell and de Groot, 1998b).

To summarize, in non-translators L1 reading is faster than L2 reading, and translation performance may be sensitive to directionality (BT vs. FT). Also, lexical access is modulated by the stimuli’s concreteness level (abstract vs. concrete words) and cognate status (cognate vs. non-cognate words). Results related to the latter two variables have been inconsistent, arguably as a consequence of lurking variables. We will contend that one such overlooked variable may be translation expertise, in line with claims that untrained translation is radically different from professional translation (Neubert, 1997) and that “the level of expertise in translation affects both the process and the product of translation” (PACTE Group, 2008: 108).

The evidence above comes from studies comparing non-translators with different levels of L2 proficiency. Naturally, between-group differences in translation tasks have been explained in terms of such a variable (de Groot and Poot, 1997; Talamas et al., 1999; Choi, 2005; Kroll and Tokowicz, 2005; Guasch et al., 2008; Dimitropoulou et al., 2011). However, these studies have failed to consider a key, distinct factor that may also underlie their results, namely, *translation expertise*. This construct has been defined as the underlying system of knowledge and skills needed to engage in successful translation (PACTE Group, 2000). It follows that the level of translation expertise depends critically on the amount of experience and competence in translation, in addition to linguistic abilities in L1 and L2 separately (see questionnaires in the Appendix). Moreover, this definition implies that translation expertise may be developed even in the absence of field-specific training, although formal translation practice is likely to bring about distinct cognitive strategies (Neubert, 1997; PACTE Group, 2000, 2008). Theoretical models of translation acknowledge that translation skills are separate from L2 proficiency (Obler, 1983; PACTE Group, 2000). Moreover, converging evidence from aphasiological (Paradis, 1984, 1994), electrostimulation (Borius et al., 2012), and neuroimaging (García, 2013) studies indicates that the neural pathways involved in translation are distinct from those involved in single-language lexical processing. It follows that the strength of semantic and form-level links between equivalents may (partially) depend on translation ability as a variable separate from L2 proficiency.

Translation expertise enhances various aspects of bilingual linguistic processing. Professional translators/interpreters outperform non-translators and/or translation students on several language-related tasks, such as semantic error detection (Fabbro et al., 1991; Yudes et al., 2012), reading speed, lexical decision on non-words, and categorization of non-typical exemplars (Bajo et al., 2000). Some of these linguistic processing advantages seem to develop during the early stages of formal translation training (Fabbro and Darò, 1995; Bajo et al., 2000) – for a review, see García (2014).

So far, only two studies have directly addressed the impact of translation ability on word reading and translation. Ibáñez et al. (2010) administered two self-paced reading tasks to both professional translators and non-translators. While the two groups showed a consistent advantage of L1 over L2, cognate effects were sensitive to translation expertise and task demands. For their part, in a word translation task, Christoffels et al. (2006) found that professional interpreters were faster than bilingual university students in both BT and FT, but that their performance was similar to that of foreign-language teachers. Whereas only the students showed a directionality effect (FT faster than BT), all three groups responded faster to cognates than non-cognates in both directions.

These findings suggest that translation expertise modulates translation directionality effects but not word-reading asymmetries ^1^. The present paper seeks to answer three related questions: are such effects, or lack thereof, sensitive to varying levels and types of translation expertise –namely, informal (non-training-based) and formal (training-based)? How are cognate and concreteness effects modulated by each type and level of translation expertise? And how soon after onset does formal translation training modulate lexical access?

To answer these questions, and considering that translation expertise can be developed even in the absence of formal training, we conducted two separate experiments. In Experiment 1, we administered two word-reading and two word-translation tasks to two groups of non-translators differing in informal translation expertise and L2 proficiency. In Experiment 2, the same tasks were performed by three groups with different levels of formal translation expertise, namely beginner translation students, advanced translation students, and professional translators.

In light of previous findings, we predicted that (i) word-reading would be faster than word-translation, and also faster in L1 than in L2, regardless of the level and type and translation expertise; (ii) overall directionality effects (BT faster than FT) would emerge only in non-translators; and (iii) RTs in both reading and translation would significantly decrease as translation expertise –be it formal or informal– increases. We also aimed to explore concreteness and cognate status effects in both types of tasks, and to establish how soon after the onset of formal training these effects emerge. More generally, if differences between groups can be explained by both L2 proficiency and translation expertise, then the current notion that only the former variable contributes to those differences is incomplete. Furthermore, the evidence for a distinctive role of translation expertise in bilingual lexical processing would be even stronger if differences emerge between samples with differing levels of translation expertise but similar levels of L2 proficiency.

## EXPERIMENT 1

### MATERIAL AND METHODS

#### Participants

Twenty-one (14 female, 7 male) adult (mean age = 34.8, SD = 18.7) English-speaking subjects who spoke Spanish as L2 participated voluntarily in this experiment. All participants were American and they were living in Tennessee at the time of testing. All had normal or corrected-to-normal vision. The subjects were late bilinguals, having learned their L2 through formal instruction. Their age of acquisition (AoA) ranged from 12 to 40 (*M* = 16.4, SD = 6.7). None of the participants possessed any field-specific training on translation. The subjects were organized in two groups. Group 1 (LOW) consisted of 11 students of Spanish with low (informal) translation expertise. Group 2 (HI) comprised 10 Spanish teachers with high (informal) translation expertise. All subjects filled in a questionnaire including demographic questions and self-rating items to assess their language and translation history. The main variables included in the questionnaire were age, AoA, L2 learning method, years of study of/exposure to the L2, L1 proficiency, L2 proficiency, BT proficiency, and FT proficiency (see Appendix 1). Both groups rated themselves as more competent in L1 than L2, and in BT than FT. **Table 1** summarizes the most relevant data.

**Table 1:** Language and translation history data of subjects in Experiment 1.

Group	Age	AoA	informal trans. exp. in years *	L1 comp **	L2 prof. ***	BT comp. **	FT comp. **	BT hs p/day	FT hs p/day
LOW *n*= 11	*M*= 19.4 (0.6)	*M*= 13.9 (1.8)	*M*= 0 (0)	*M*= 6.9 (0.3)	*M*= 4.0 (0.7)	*M*= 3.8 (0.7)	*M*= 3.7 (0.6)	*M*= 1.3 (0.5)	*M*= 1.3 (0.5)
HI *n*= 10	*M*= 51.8 (13.0)	*M*= 19.2 (8.9)	*M*= 0 (0)	*M*= 6.7 (0.6)	*M*= 5.4 (1.1)	*M*= 5.7 (1.0)	*M*= 5.1 (1.1)	*M*= 1.3 (0.6)	*M*= 1.4 (0.6)

*‘Translation experience in years’ includes years as a translation student and as a practicing professional.

**‘Comp.’, Competence.

***‘Prof.’, Proficiency.

*Standard deviations are provided within parentheses*.

*Competence and proficiency were self-rated by subjects on a 7 point-scale (1 = none; 7 = optimal)*.

*BT and FT hours per day: 1 = less than 1; 2 = from 1 to 4; 3 = from 4 to 10*.

The Mann-Whitney independent-samples *U* test revealed that HI had a higher rank than LOW in age (*p* < 0.001), L2 proficiency (*p* = 0.005), BT competence (*p* = 0.001), and FT competence (*p* = 0.005). There were no significant differences between groups in the distribution of the variables AoA, and L1 competence (Independent-samples Mann-Whitney *U* test, *p* > 0.05). There were no significant differences in daily practice time in BT (independent-samples Mann-Whitney *U* test, *p* = 0.654) or in FT (independent-samples Mann-Whitney *U* test, *p* = 0.918).

#### Stimuli

One hundred and 92 semantically equivalent noun pairs were constructed. Spanish stimuli were distributed in three 64-item blocks (SP1, SP2, SP3), as were the English stimuli (EN1, EN2, EN3). Blocks SP1, SP2, EN1, and EN2 were used for translation tasks, whereas blocks SP3 and EN3 were used for reading tasks. Thus, in controlling for stimulus variables across tasks, blocks SP1 and SP2 were merged into a single translation block (SPT), whereas blocks EN1 and EN2 were merged into another large translation block (ENT).

The items in each English block were matched to their corresponding Spanish block. Each of the six blocks contained 16 concrete cognates (e.g., *paper*, *papel*), 16 abstract cognates (e.g., *comedy*, *comedia*), 16 concrete non-cognates (e.g., *table*, *mesa*), and 16 abstract non-cognates (e.g., *punishment*, *castigo*; (see Appendix 3). Statistical comparisons of syllabic length between all blocks yielded no significant differences (SPT vs. ENT: *p* = 0.99; SP3 vs. EN3: *p*= 0.99; SPT vs. SP3: *p*= 1; ENT vs. EN3: *p*= 0.99). Similarly, all blocks were matched for rank (order of appearance in the corresponding corpus) within and between languages (SPT vs. ENT: *p* = 0.97; SP3 vs. EN3: *p*= 0.99; SPT vs. SP3: *p*= 0.99; ENT vs. EN3: *p*= 0.98). Also, blocks belonging to the same language were matched for frequency (SPT vs. SP3: *p*= 0.95; ENT vs. EN3: *p*= 0.98). All rank and frequency data for both languages were obtained from Davies (2008a,b).

To avoid participant-strategy artifacts, stimuli were first randomly distributed within each block. Importantly, semantically related nouns were at least five items apart from each other, which reduced the possibility of categorical interference (Kroll and Stewart, 1994).

#### Design and procedure

Participants were tested individually in a dimly illuminated room. They were asked to sit behind a desk facing a computer screen. Instructions and stimuli were delivered using custom software developed in the Python programming language ^2^ with the Pygame development library ^3^ This software also recorded the participants’ RTs and relevant information about the tasks. Oral instructions prior to the tasks and on-screen written instructions during the tasks were provided in English. An examiner monitored the participants’ performance.

Each participant performed four tasks, namely L1 reading (L1R), L2 reading (L2R), BT, and FT. The tasks were counterbalanced across participants, so that no two subjects performed them in the same order. All reading tasks (L1R, L2R) used blocks EN3 and SP3. The other blocks (EN1, EN2, SP1, SP2) were alternated within-subjects so that the equivalent pairs in BT were not the same as those used in FT (if a subject performed BT with block SP1, then FT was performed with block EN2). This prevented cross-language priming effects between tasks.

Participants were instructed to either read out loud (in L1R and L2R) or translate (in BT and FT) the words appearing on the screen as fast and accurately as possible. Each trial began with an ocular fixation cross at the center of the screen, appearing 300 ms before the display of each word. The words appeared in white letters (font: Times New Roman; size: 70) against a black background in the middle of the screen. The words remained on the screen for another 200 ms. Participants were asked to press a key as soon as they were ready to articulate their response. The keystroke served both to record RTs and to cue the following trial. Immediately after each keystroke, participants pronounced their response out loud (the inter-trial interval was long enough to prevent overlap between successive responses). As the task progressed, the examiner completed a control grid discriminating valid and invalid responses (exclusion criteria are detailed in the Results section). Tasks were separated by a two-minute break. The complete session for each participant lasted roughly 30 min.

The use of a manual response to measure RTs deviates from standard practice in the field, which involves the use of a voice-activated switch. Our reason to choose this procedure was methodological in nature. RTs in oral responses are affected by variables such as the manner of articulation (e.g., fricative, plosive) of the initial phoneme and the structure of the initial syllable (e.g., consonant-vowel, vowel-consonant, cluster-consonant; Rastle and Davis, 2002; Rastle et al., 2005). It was not possible to control for these variables in addition to the ones already contemplated, namely, word class, length, rank, frequency, cognate status, and concreteness. Thus, manual responses offered a viable alternative, as they are unaffected by such phonological effects. Importantly, mean RTs in our experiments were similar to those obtained via voice-activated switches in previous studies, and their modulation by our target variables resembled previous findings in the literature (see below). Also note that vocal and manual responses were equally sensitive to lexical effects in other linguistic tasks –e.g., word frequency in Hutson et al. (2011).

#### Statistical analysis

Invalid responses were eliminated for analysis, but their proportion was previously compared across expertise levels with a Chi square test. Valid data were log transformed to make them amenable to parametric analysis.

Within each participant, log (RT) were averaged for each combination of task, cognate status, and concreteness. Those averages were analyzed using a four-way mixed-model analysis of variance (ANOVA) with a between-subject factor with 2 levels of informal translation expertise (level: LOW, HI) and 3 within-subjects factors with different levels: task (L1R, L2R, BT, FT), cognate status (Cog, NCog), and concreteness (Abs, Con). The Tukey *a posteriori* test was used to examine the pairwise comparison for significant ANOVAs. Given the large number of pairwise comparisons, *p*-values for interactions are reported in the supplementary material (Appendix 4). All analyses were performed using Statistica 10.0 (Statsoft).

### RESULTS

#### Invalid responses

Five exclusion criteria were considered: (i) no response (e.g., subject remains silent); (ii) hesitation or false start (e.g., *fury*→*fueg… furia!*); (iii) task confusion (e.g., subject reads when asked to translate, or vice versa); (iv) wrong translation (e.g., *fury*→*fuera*); and (v) non-predefined translation (e.g., *fury*→ *ira*). ^4^

Across participants, invalid responses varied from 5 (2.0) to 72 (28.1%). The proportion of invalid responses was inversely related with proficiency level (LOW = 22.4%, HI = 8.0%; Chi square (1) = 213.931, *p* < 0.001; Gamma = 0.539, *p* < 0.001). As measured by *post hoc* Tukey tests, LOW had a significantly larger number of invalid trials on L2R (*p* = 0.001), BT (*p* < 0.001), and FT (*p* < 0.001). No significant level differences were found in L1R.

#### Reaction times

***ANOVA: Main effects***. There was no significant difference [*F* (1,19) = 0.08, *p* = 0.778, partial η^2^ = 0.004) in RT between the groups. Across tasks, all RTs were significantly different [*F*(3,57) = 239.12, *p* < 0.001, partial η^2^ = 0.926]. According to the *a posteriori* analysis (Tukey’s HSD test, MSe = 0.045, df = 57), the rank order of the means increased as follows: L1 < L2 < BT < FT (all significant *p*s < 0.001). Concrete words produced significantly shorter RTs than abstract words [*F* (1,19) = 20.30, *p* < 0.001, partial η^2^ = 0.516]. RTs for cognates were significantly shorter than for non-cognates [*F*(1,19) = 90.78, *p* < 0.001, partial η^2^ = 0.827]. See Supplementary Data for (Appendix 4) further statistical details. All these main effects were nuanced by the presence of significant interactions, as described below.

***ANOVA: interaction effects***. We found four two-way and two three-way significant interactions. Five of them involved the task performed. All these interactions were scrutinized with the Tukey *a posteriori* test.

**FIGURE 1:**
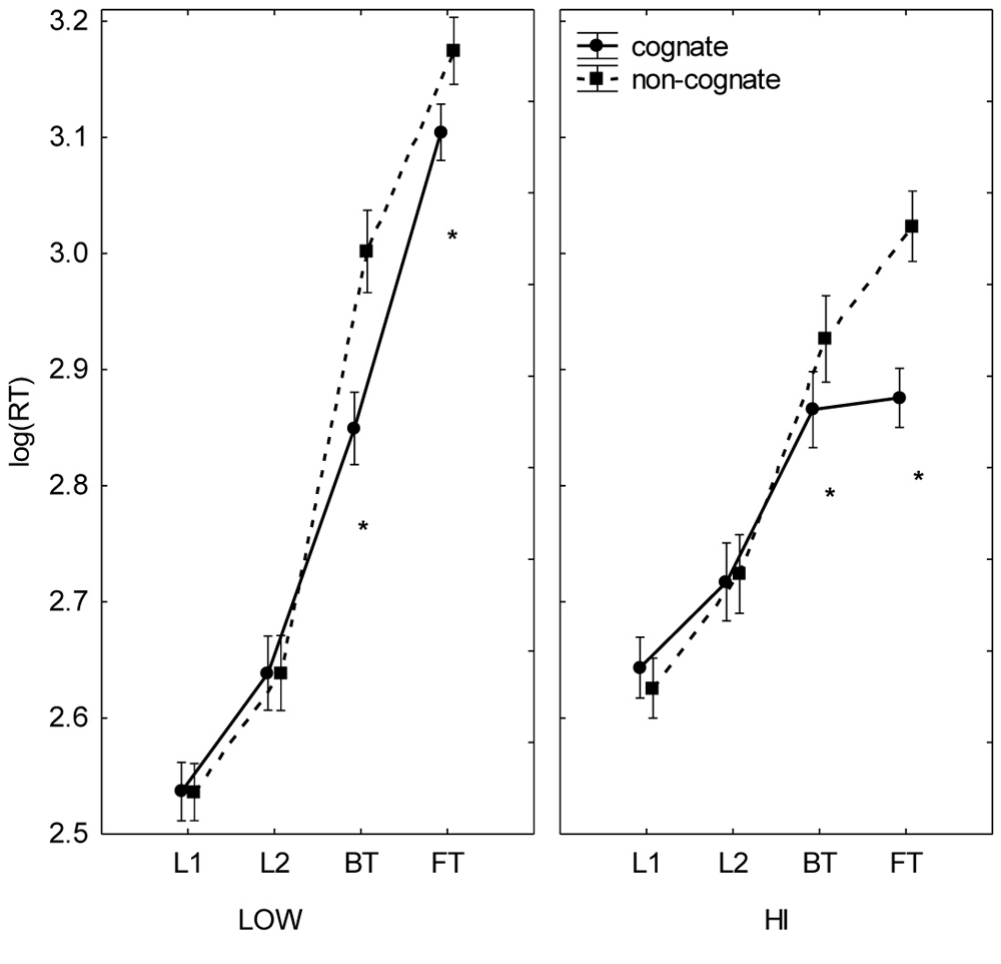
**Cognate effects in English–Spanish bilinguals with different levels of informal translation competence.** Mean log (RT) by proficiency level (LOW, HI), task (L1R, L2R, BT, FT), and cognate status (cognate, non-cognate). Bars denote one SE above and below the mean. Asterisks indicate significant differences between cognate and non-cognate stimuli.

**FIGURE 2:**
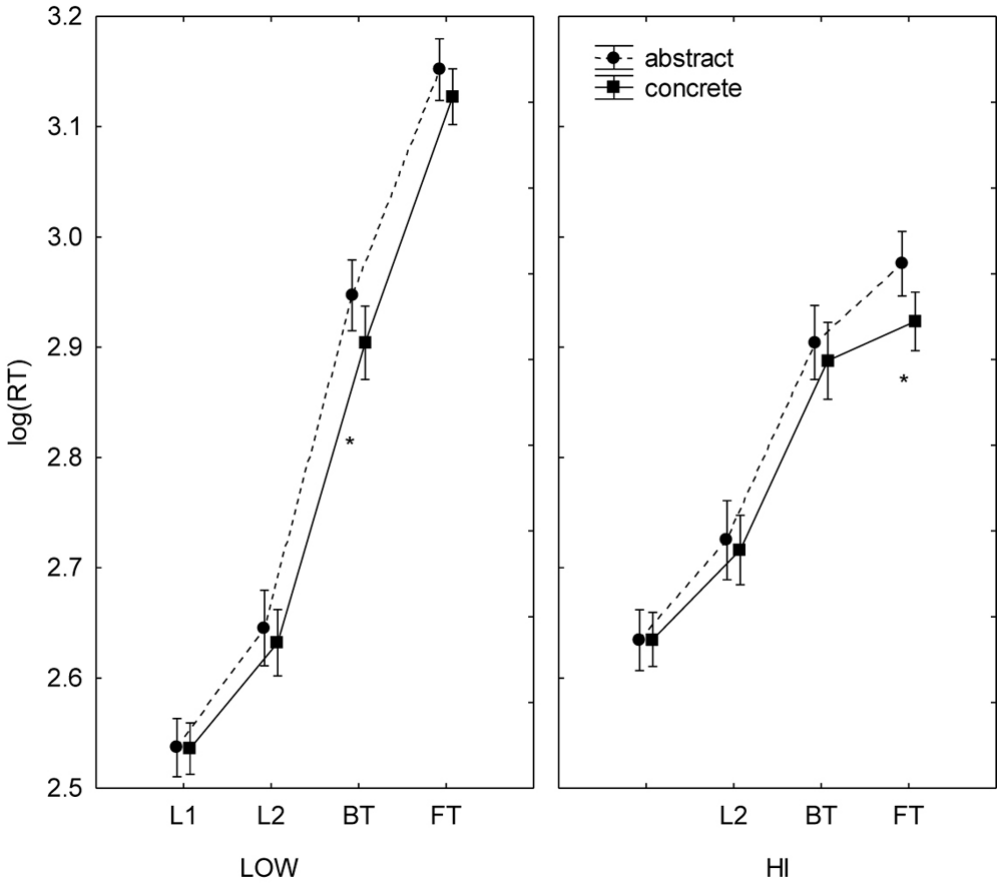
**Concreteness effects in English–Spanish bilinguals with different levels of informal translation competence.** Mean log (RT) by proficiency level (LOW, HI), task (L1R, L2R, BT, FT), and concreteness (concrete, abstract). Bars denote one SE above and below the mean. Asterisks indicate significant differences between concrete stimuli and abstract.

The effect of task interacted with that of competence [*F*(3,57) = 23.86, *p* < 0.001, partial η^2^ = 0.557); the LOW group showed the same differences between tasks as reported in the main effects (L1 > L2 > BT > FT), but the HI group showed differences between reading (faster) and translation (slower) tasks, but not within them. The effect of task interacted with the words’ cognate status [*F*(3,57) = 52.88, *p* < 0.001, partial η^2^ = 0.736]; cognates were processed faster than non-cognates in translation, but not in reading (**Figure 1**). Similarly, the effect of task also interacted with concreteness [*F*(3,57) = 4.82, *p* < 0.001, partial η^2^ = 0.202); concrete words were processed faster than abstract words only in the translation tasks (**Figure 2**). Cognate status and concreteness also interacted in their effects [*F*(1,19) = 10.67, *p* = 0.004, partial η^2^ = 0.360]; concrete words were processed significantly faster than abstract words only in the non-cognate condition.

The variables of task and cognate status also appeared in two three-way interactions. First, the interaction between task, cognate status, and level of competence [*F*(3,57) = 15.70, *p* < 0.001, partial η^2^ = 0.452] showed that the translation directionality effect (BT faster than FT) was present for non-cognates in both levels, but for cognates only in LOW. A significant difference between levels was also observed; in FT, the HI group processed cognates faster than the LOW group (**Figure 1**). Second, task, cognate status, and concreteness interacted [*F*(3,57) = 4.47, *p* = 0.007, partial η^2^ = 0.191] as follows: the differences in RTs in terms of cognate status (cognate <non-cognate) and concreteness (concrete < abstract) were significant in the translation but not in the reading tasks. See Supplementary Data (Appendix 4) for further statistical detail of these interaction effects.

Finally, we conducted an additional analysis to confirm that our methodological procedure was unaffected by phonological variables shown to modulate vocal RTs (Rastle and Davis, 2002; Rastle et al., 2005). To this end, we analyzed the groups’ (log)RTs in the BT task on three sub-lists of English responses (target words) differing in their initial phoneme type (12 plosive-initial words, 12 fricative-initial words, and 12 vowel-initial words). The three sub-lists were matched for syllabic length [*F*(2,33) = 0.006, *p* = 0.994] and frequency [*F*(2,33) = 0.002, *p* = 0.998]. We conducted a mixed-model ANOVA with initial phoneme type as a within-subject factor and (informal) translation expertise as a between-subject factor. There was no significant effect of either initial phoneme type [*F*(2,38) = 2.05, *p*= 0.143, partial η^2^ = 0.097] or expertise [*F*(1,19) = 0.06, *p*= 0.816, partial η^2^ = 0.003] in log(RT). There was no significant interaction between these variables [*F*(2,38) = 3.02, *p*= 0.061, partial η^2^ = 0.137]. These tests suggest that our results were not affected by uncontrolled sublexical variables and that the lag between each click and its subsequent utterance was fairly constant.

In sum, although LOW and HI had similar response latencies collapsing all conditions, they differed in some important aspects. While reading was faster than translation for both groups, asymmetries within each task type were found only in LOW (L1R faster than L2R, BT faster than FT). For HI, reading and translating were not significantly faster in either language or direction, respectively. Also, both groups showed cognate and concreteness effects, but only in the translation tasks. Cognates and concrete nouns were translated faster than non-cognates and abstract nouns, respectively. The concreteness effect, however, affected only non-cognates. Finally, whereas both groups translated non-cognates similarly (faster in BT than in FT), they differed in their processing of cognates. LOW translated cognate items faster in BT than in FT, but such asymmetrical processing of cognates disappeared in HI. The only significant inter-group differences between translation directions involved cognates in FT. In general terms, these results are in line with previous findings in the literature.

### DISCUSSION OF EXPERIMENT 1

The results of Experiment 1 are in line with previous findings in studies with non-translators. However, our HI group had significantly higher ratings than LOW not just on L2 proficiency, but also on BT and FT competence. This suggests that between-group differences usually explained in terms of L2 proficiency may also be reflecting differences in translation-specific skills. Incidentally, the replication of both main and interaction effects reported in the literature attests to the suitability of our experimental procedure.

#### Word reading

Word reading was faster in L1 than in L2 only for LOW, which suggests that, at low proficiency levels, the links supporting lexical recognition and production are stronger for native than non-native languages (de Groot et al., 1994; Kroll and Stewart, 1994; Dijkstra and van Heuven, 2002). On the other hand, reading latencies were similar for both languages in HI, corroborating that asymmetries between languages attenuate as proficiency increases (Kroll and Stewart, 1994; Kroll et al., 2002; Schweiter and Sunderman, 2009; Kroll et al., 2010). However, it is likely that such patterns reflect language-dominance rather than just age-of-acquisition effects (Heredia, 1997; see also Gayane and Hernández, 2006).

These differences notwithstanding, both groups were similar in that reading was faster than translation overall, as previously observed by Kroll and Stewart (1994) and La Heij et al. (1996). Also, the lack of concreteness effects in the reading conditions confirms that word naming does not require access to the conceptual level (Duyck and Brysbaert, 2004, 2008). Finally, the absence of a cognate effect in the reading tasks may be explained as follows. When a word recognized, its receptive orthographic and/or phonological representations co-activate their counterparts in the production system. Since the correspondence of form representations between receptive and productive vocabulary *of the same language* is always total, then cognates have no processing advantage over non-cognates during word reading. ^5^ The situation is different for interlinguistic processes, as explained below.

#### Word translation

Overall translation directionality effects were observed in LOW but not in HI, which is consistent with the view that translation asymmetries attenuate as L2 proficiency increases (Talamas et al., 1999). However, our results suggest that such between-group discrepancies may also be reflecting differences in translation competence (see General Discussion). Additionally, the groups’ translation performance was modulated by word variables.

***Semantic effects***. Indicative of semantic involvement, the concreteness effect consisted in a significant advantage of concrete over abstract nouns, as previously found by de Groot (1992), de Groot et al. (1994), and van Hell and de Groot (1998b). A plausible explanation is that concrete translation equivalents share more semantic features than abstract words, whose meanings are more diffuse and typically include more language-specific connotations. The activation of shared features during source-language word processing facilitates access to its target-language counterpart. Since concrete equivalents have more semantic representations in common than do abstract equivalents, the former are translated faster (van Hell and de Groot, 1998a,b; Duyck and Brysbaert, 2008).

Such an effect was not modulated by translation direction, as shown in previous translation experiments using color terms (La Heij et al., 1996), number words (Duyck and Brysbaert, 2004), and concrete nouns (de Groot et al., 1994). Moreover, this finding suggests that the impact of semantic variables is not influenced by either L2 proficiency (de Groot and Comijs, 1995; de Groot and Poot, 1997; Duyck and Brysbaert, 2004) or informal translation competence. Finally, the presence of concreteness effects in both translation directions contradicts the view that BT must bypass semantic access (Kroll and Stewart, 1994; Sholl et al., 1995). For further evidence that both translation directions may be semantically mediated, see de Groot et al. (1994), de Groot and Comijs (1995), de Groot and Poot (1997), Duyck and Brysbaert (2004).

***Form-related effects***. We also found form-related effects, as cognates were translated significantly faster than non-cognates. A widely accepted interpretation of this phenomenon is that words in the bilingual lexicon are activated in a parallel, language-non-selective fashion. When a word is processed in one language, a cohort of related words in the other language is co-activated, especially if they share orthographic, phonological, or semantic properties. Both non-cognate and cognate equivalents share semantic information, but since only the latter share graphemic and/or phonological attributes, they induce additional facilitation at the form level (van Hell and de Groot, 1998a,b; Duyck and Brysbaert, 2004, 2008).

We also found that both groups translated non-cognates faster in BT than in FT. This suggests that the relative contributions of form- and meaning-based connections for equivalents with little or no orthographic/phonological overlap are not significantly modulated by either L2 proficiency or informal translation competence. However, a directionality effect for cognates was observed in LOW but not in HI, as reported elsewhere in the literature (de Groot et al., 1994, Experiment 1; Sánchez-Casas et al., 1992). Building on the finding that cognate translation depends more on form-level processing than does non-cognate translation, one explanation for this effect is that direct word–word connections are stronger for BT than FT at low levels of proficiency, but that such asymmetries disappear with increasing levels of L2 proficiency (de Groot et al., 1994; Talamas et al., 1999; Kroll et al., 2010) ^6^ and/or informal translation competence.

#### Summary

The present results reveal processing differences between the groups, the tasks, and the influence of concreteness and cognate status on them. Also, they suggest that a variable that has been heretofore overlooked bilingual memory studies, namely translation competence, may play a role in such differences. Specifically, the level of translation competence, as a variable that is distinct from L2 proficiency, may influence the strength of inter-equivalent links. To further explore this factor, in Experiment 2 we used the same paradigm with three groups of bilinguals possessing varying levels of formal (training-based) translation expertise.

## EXPERIMENT 2

### METHOD

#### Participants

Thirty-six (27 female, 9 male) adult (mean age = 26.5, SD = 7.7) Spanish-speaking subjects who spoke English as L2 participated voluntarily in this experiment. All participants were Argentinean. All had normal or corrected-to-normal vision. Except for three subjects, all were late bilinguals, having learned their L2 through formal instruction. Their AoA ranged from 5 to 21 (*M*= 9.5, SD = 3.3). The subjects were organized in three groups. Group 1 (BEG) consisted of 12 highly proficient bilinguals who were beginner students at an undergraduate program in translation. Since all but one of them were freshmen at the beginning of the academic year, their level of translation expertise was assumed to range from minimal to null. Group 2 (ADV) comprised 12 high-proficiency bilinguals who were in their senior year at the same translation program. Group 3 (PRO) was made up of 12 professional translators with at least 3 years of experience in the field. All subjects filled in a questionnaire including demographic questions and self-rating items to assess their language and translation history. The variables considered were the same as those included in the questionnaire used in Experiment 1 (see Appendix 2). All three groups rated themselves as more competent in L1 than L2, and in BT than FT. **Table 2** below summarizes the most relevant data.

**Table 2:** Language and translation history data of subjects in Experiment 2.

Group	Age	AoA	Formal trans. exp. in years *	L1 comp. **	L2 prof. ***	BT comp. **	FT comp. **	BT hs p/day	FT hs p/day
BEG *n*= 12	*M*= 19.2 (1.4)	*M*= 9.0 (2.6)	*M*= 1.0 (0.4)	*M*= 6.1 (0.9)	*M*= 5.1 (0.8)	*M*= 4.3 (1.7)	*M*= 3.9 (0.9)	*M*= 1.4 (0.5)	*M*= 1.4 (0.5)
ADV *n*= 12	*M*= 25.6 (3.8)	*M*= 8.91 (2.9)	*M*= 4.0 (0.7)	*M*= 6.5 (0.5)	*M*= 5.8 (1.0)	*M*= 5.1 (1.0)	*M*= 4.7 (1.0)	*M*= 1.2 (0.4)	*M*= 1.2 (0.4)
PRO *n*= 12	*M*= 34.7 (6.5)	*M*= 10.6 (4.3)	*M*= 12.6 (3.9)	*M*= 6.6 (0.6)	*M*= 6.1 (0.7)	*M*= 6.2 (0.6)	*M*= 5.8 (0.8)	*M*= 1.8 (0.9)	*M*= 1.5 (0.9)

*‘Translation experience in years’ includes years as a translation student and as a practicing professional.

**‘Comp.’ = Competence.

***‘Prof.’ = Proficiency.

*Standard deviations are provided within parentheses*.

*Competence and proficiency were self-rated by subjects on a 7 point-scale (1 = none; 7 = optimal)*.

*BT and FT hours per day: 1 = less than 1; 2 = from 1 to 4; 3 = from 4 to 10*.

The Kruskal–Wallis independent-samples test revealed significant differences between groups in the distribution of age (*p* < 0.001), TR training (*p* < 0.001), L2 proficiency (*p* = 0.023), BT competence (*p* = 0.001), and FT competence (*p* < 0.001). Using the Mann-Whitney independent-samples *U* test between all pairs of groups, with sequential Bonferroni correction, the results are as follows. For age and translation expertise, the rank differences were significant between all pairs (all corrected *p*s < 0.001), the order being: PRO > ADV > BEG. For L2 proficiency, the only significant rank difference (*p* < 0.001) was between PRO (*M*= 6.1, SD = 0.7) and BEG (*M*= 5.1, SD = 0.8). For BT competence and FT competence, PRO had a significantly higher rank than BEG and ADV (*p* < 0.01), but there was no difference between the latter two groups (*p* > 0.05). There were no significant differences between groups in the distribution of the variables AoA and L1 competence (Independent-samples Kruskal–Wallis test, *p* > 0.05). There were no significant differences in daily practice time in BT (Independent-samples Kruskal–Wallis test, *p* = 0.233) or in FT (Independent-samples Kruskal–Wallis test, *p* = 0.660).

In sum, BEG and ADV were similar in L2 proficiency, but ADV had a significantly higher rating in translation expertise. L2 proficiency was also similar between ADV and PRO, but PRO had significantly higher ratings than ADV in translation expertise, BT competence, and FT competence. Thus, whatever differences are observed between BEG and ADV or between ADV and PRO will be reflecting the influence of translation-specific skills rather than that of L2 proficiency.

#### Stimuli

The same stimuli blocks described for Experiment 1 were used in this second experiment. The only difference was that BT tasks used English blocks (either EN1 or EN2), whereas FT used Spanish blocks (either SP1 or SP2).

#### Design and procedure

The design and procedure in Experiment 2 were exactly the same as those in Experiment 1.

#### Statistical analysis

The analysis strategy in Experiment 2 was the same as that in Experiment 1, except that in this case there were only three levels of (formal) translation expertise (BEG, ADV, and PRO) as a between-subject factor. The Tukey *a posteriori* test was used to examine the pairwise comparison for significant ANOVAs. Given the large number of pairwise comparisons, *p*-values for interactions are reported in the supplementary material (Appendix 4). All analyses were performed using Statistica 10.0 (Statsoft).

### RESULTS

#### Invalid responses

Response exclusion criteria were the same as those enumerated for Experiment 1. Across participants, invalid responses ranged from 15 (5.9) to 64 (25.0%). The proportion of invalid responses was inversely related with translation expertise (BEG = 18.3, ADV = 13.3, PRO = 10.8%; Chi square (2) = 73.326, *p* < 0.001; Gamma = 0.205, *p* < 0.001). A *post hoc* Tukey test revealed no differences between level pairs on L1R and L2R. However, the proportion of invalid responses was significantly larger for BEG than for PRO in BT (*p* < 0.001), and for BEG than for ADV (*p* = 0.029) and PRO (*p* < 0.001) in FT. There were no significant differences between ADV and PRO in either BT or FT.

#### Reaction times

***ANOVA: main effects***. There was a significant effect of level of expertise [*F*(2,33) = 4.50, *p* = 0.019, partial η^2^ = 0.214] in mean log (RT). The *a posteriori* analysis (Tukey’s HSD test, *MSe* = 0.111, *df* = 33) revealed a shorter reaction time in ADV (*p* = 0.041) and PRO (*p* = 0.032) relative to BEG. ADV and PRO did not differ from each other (*p* > 0.05). Across tasks, RTs were significantly different [*F*(3,99) = 218.08, *p* < 0.001, partial η^2^ = 0.869]. According to the *a posteriori* analysis (Tukey’s HSD test, MSe = 0.015, df = 33), the rank order of the means increased as follows: L1 < L2 < BT = FT (all significant *p*s < 0.001). Concrete words took less time to process than abstract ones [*F*(1,33) = 40.40, *p* < 0.001, partial η^2^ = 0.550]. Finally, RTs were significantly shorter for cognates than for non-cognates [*F*(1,33) = 343.97, *p* < 0.001, partial η^2^ = 0.912]. See Supplementary Data (Appendix 4) for further statistical detail of these main effects.

***ANOVA: interaction effects***. We found three two-way and three three-way significant interactions. Five of them involved the task performed. All interactions were scrutinized with the Tukey *a posteriori* test.

**FIGURE 3:**
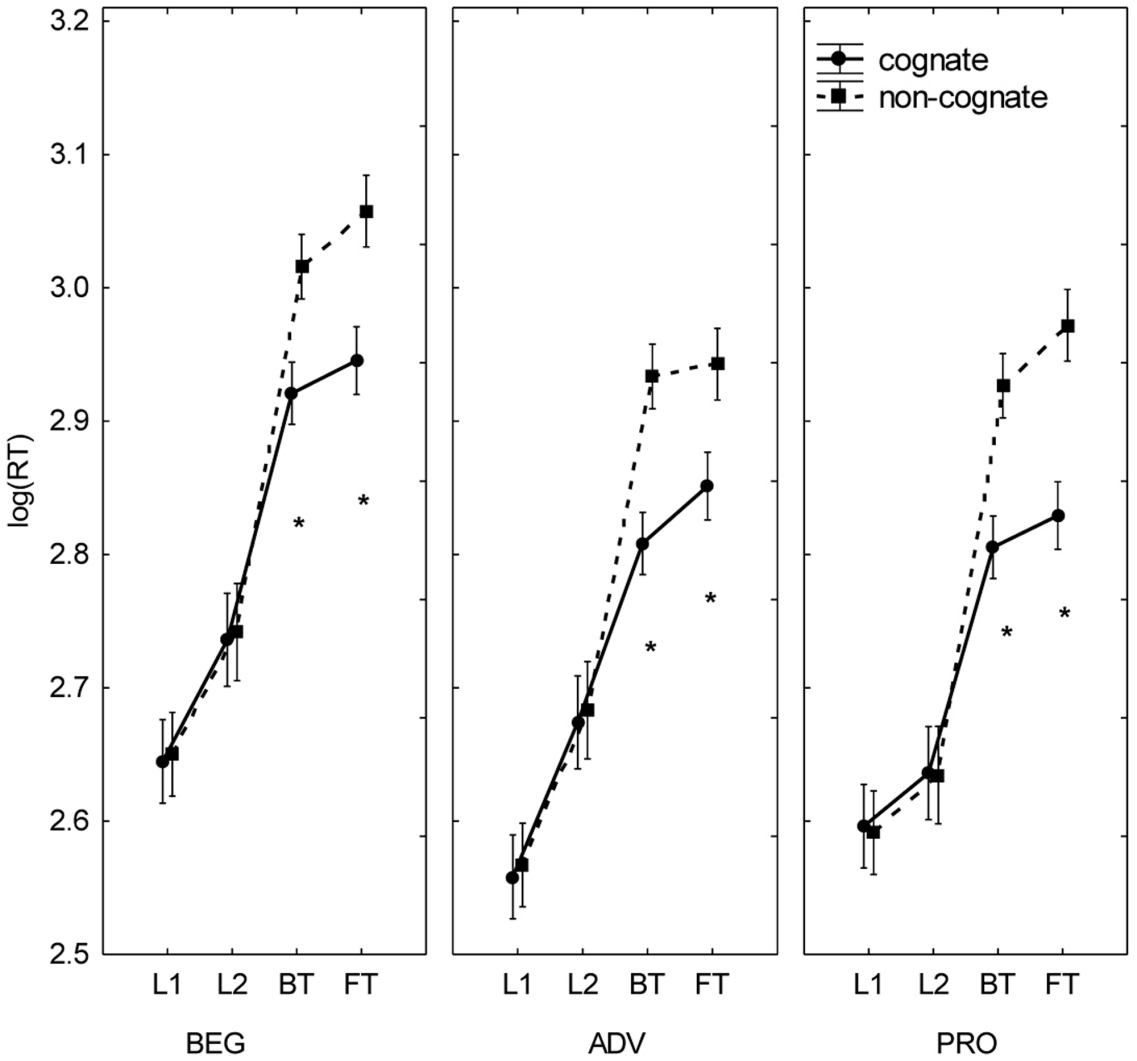
**Cognate effects in Spanish–English translators with different levels of formal translation expertise.** Mean log (RT) by expertise level (BEG, ADV, PRO), task (L1R, L2R, BT, FT), and cognate status (cognate, non-cognate). Bars denote one SE above and below the mean. Asterisks indicate significant differences between cognate and non-cognate stimuli.

As in Experiment 1, the task by concreteness interaction [*F*(3,99) = 9.37, *p* < 0.001, partial η^2^ = 0.221] revealed that concrete words were processed faster than abstract words in translation but not in reading (**Figure 3**). Similarly, the task by cognate status interaction [*F*(3,99) = 141.42, *p* < 0.001, partial η^2^ = 0.811] showed a cognate effect only in the translation tasks, with shorter RTs for cognates than non-cognates. A final two-way interaction was found between cognate status and concreteness [*F*(1,33) = 6.81, *p* = 0.014, partial η^2^ = 0.171]. The Tukey test found all comparisons significant, so it is not easy to propose a clear interpretation of this interaction without considering their qualification by level (see below).

**FIGURE 4:**
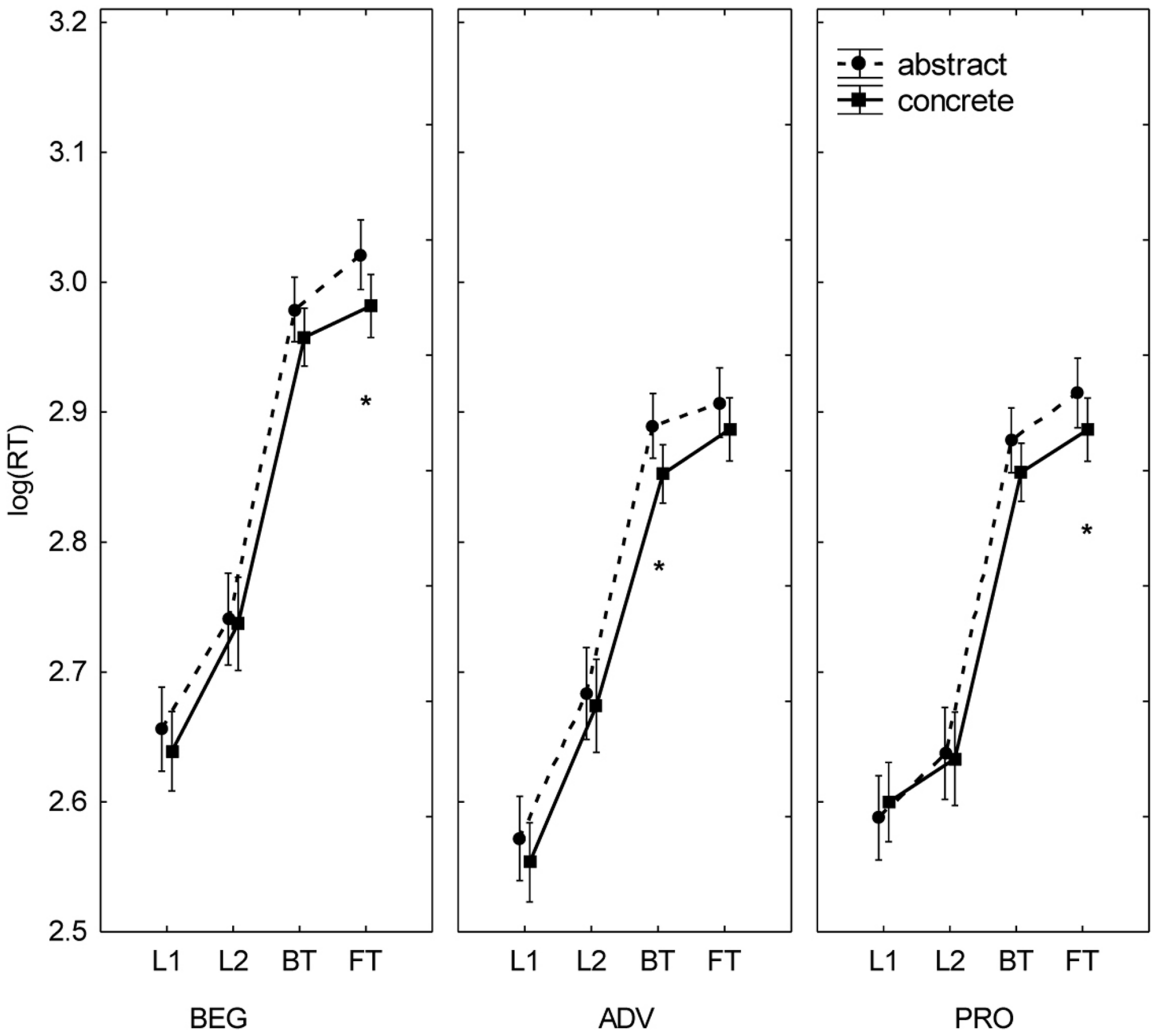
**Concreteness effects in Spanish–English translators with different levels of formal translation expertise.** Mean log (RT) by expertise level (BEG, ADV, PRO), task (L1R, L2R, BT, FT), and concreteness (concrete, abstract). Bars denote one SE above and below the mean. Asterisks indicate significant differences between concrete and abstract stimuli.

Analysis of the three-way interactions yielded intriguing results. The interaction between task, level, and concreteness [*F*(6,99) = 2.21, *p* = 0.048, partial η^2^ = 0.118] revealed a significant directionality effect (BT faster than FT) in concrete and abstract words in PRO (**Figure 4**); in ADV, BT was faster than FT only for concrete words; and in BEG, only for abstract words. The analysis of the task by level by cognate status interaction [*F*(6,99) = 3.55, *p* = 0.003, partial η^2^ = 0.177] revealed directionality effects (BT faster than FT) within each level, but not uniformly across the stimuli’s cognate status (**Figure 3**). In BEG and PRO, BT was significantly faster than FT with non-cognates but not with cognates; in ADV, the opposite was true. Finally, a third three-way interaction was detected between task, cognate status, and concreteness [*F*(3,99) = 3.46, *p* = 0.019, partial η^2^ = 0.095]. As observed in Experiment 1, the differences in RTs in terms of cognate status (cognate < non-cognate) and concreteness (concrete < abstract) were significant in translation but not in reading. See Supplementary Data (Appendix 4) for further statistical detail of these interaction effects.

Finally, as we did in Experiment 1, we conducted an additional analysis to confirm that our methodological procedure was unaffected by phonological variables. In this case, we analyzed the groups’ (log)RTs in the FT task on the same three sub-lists of English responses (target words) used for Experiment 1. Once again, we conducted a mixed-model ANOVA with initial phoneme type as a within-subject factor and (formal) translation expertise as a between-subject factor. This time there was a significant effect [*F*(2,66) = 6.64, *p*= 0.002, partial η^2^ = 0.167] of initial phoneme type in log(RT), with vowel-initial words yielding significantly longer RTs than plosive- and fricative-initial words (Tukey’s HDS test, MSe = 0.057, df = 66). There was no significant effect of expertise [*F*(2,33) = 2.50, *p*= 0.098, partial η^2^ = 0.131], and there was no interaction between both variables [*F*(4,66) = 2.32, *p*= 0.066, partial η^2^ = 0.123]. On close inspection, the difference in initial phoneme type was attributable only to the BEG group (Tukey’s HDS test, MSe = 0.013, df = 61), since neither ADV nor PRO showed any differences among initial phoneme types. Also, note that in BEG there was no difference between plosive-initial and fricative-initial words. All in all, these analyses indicate that our results were almost completely independent from sublexical variables and that the lag between each click and its subsequent utterance was fairly constant.

In sum, collapsing all tasks, BEG was slower than ADV and PRO, but the latter two groups performed similarly. Other results replicate findings from Experiment 1. Reading was significantly faster than translation in all groups; and, as was the case with LOW in Experiment 1, reading was faster in L1 than in L2. Notice that an advantage of L1 over L2 irrespective of translation expertise has also been reported by Ibáñez et al. (2010) in their self-paced reading study. Also, neither group showed an overall directionality effect in word translation, which replicates the result obtained by Christoffels et al. (2006) with professional interpreters. Furthermore, the advantage of concrete over abstract nouns, and of cognates over non-cognates, was observed only in the translation tasks. The latter effect has also been shown to occur independently of translation expertise (Christoffels et al., 2006).

### DISCUSSION OF EXPERIMENT 2

#### Coincidences with Experiment 1

The acquisition of formal (i.e., training-based) translation expertise, to any extent, does not seem to modify general aspects of bilingual isolated-word processing, namely: (i) the greater cognitive demands of word translation as compared to word reading; (ii) the tendency for reading to be easier in L1 than in L2; (iii) the absence of semantic involvement in word reading in either language; (iv) the absence of overall translation asymmetries; (v) the greater import of word-form similarity between equivalents in translation over reading; and (vi) the prevalence of language-nonselective access. However, formal translation training does seem to have an impact on other aspects of lexical retrieval, as discussed below.

#### Between-group differences

Overall, BEG had significantly longer RTs than both ADV and PRO. Since BEG and ADV had comparable levels of L2 proficiency, their differences must be attributed to their discrepant level of translation expertise. In this sense, the differential between-group effects observed cannot be explained as a function of how many hours a day each group engages in BT or FT, since all groups had similar ratings in these variables. Instead, such differences seem to reflect the impact of years of translation experience. This implies that lexical processing speed may be influenced directly by translation expertise, irrespective of L2 proficiency. The absence of overall differences between ADV and PRO might be due to a ceiling effect, whereby lexical links within and across languages would reach their maximum strengthening at advanced, pre-professional levels of translation practice, in addition to high levels of L2 proficiency.

This pattern of results suggests that the effects of formal translation expertise might become significant shortly after the onset of translation training, as suggested by Fabbro and Darò (1995). In this sense, previous studies have shown that sophomore and even freshman interpretation students outperform non-translator bilinguals on several linguistic and executive-function tasks (Bajo et al., 2000; Köpke and Nespoulous, 2006; Tzou et al., 2011; Yudes et al., 2012). Also, Elmer et al. (2010) showed that functional and electrophysiological differences between professional interpreters and non-translators may be associated with training during translation education rather than the amount of professional translation expertise. Our results corroborate that translation training may play a distinct role in lexical processing, especially during the early stages of its development. This may be so because early translation training emphasizes specific cognitive processes. In particular, during the first months of formal translation education prospective translators establish and analyze interlinguistic associations more frequently and intensely than they did before enrolment. We speculate that continual reflection about similarities and differences between equivalents leads beginner students to recognize and reinforce novel inter-linguistic associations while inhibiting cross-language connections to representations which they wrongly believed to be shared between equivalents. This possibility, however, remains to be empirically assessed.

***Semantic effects***. Although there were no differences between BT and FT as revealed by the main effect of task, translation asymmetries did emerge for certain word types in specific groups. BT was faster than FT for abstract words in BEG, concrete words in ADV, and both types in PRO. In Experiment 1, the concreteness effect was not modulated by translation direction, which suggests that the pattern observed in Experiment 2 depends more on the type of translation expertise than on L2 proficiency. A tentative explanation is that the semantic links between translation equivalents reconfigure their strengths as formal translation expertise increases. This view is consistent with previous behavioral evidence of specifically semantic effects caused by translation/interpreting training (Fabbro et al., 1991; Bajo et al., 2000; Yudes et al., 2012) and with neurophysiological data showing that professional interpreters feature “a training-induced altered sensitivity to semantic processing within and across L1 and L2” (Elmer et al., 2010: 152).

The fact that all three groups manifested their respective concreteness-related effects as an advantage of BT over FT might reflect their greater competence in translating into L1 than into L2 (see Participants above). However, we lack a theoretical rationale to account for the finding that, in BT, BEG, and ADV showed an advantage of abstract and concrete words, respectively. The fact that no such effects were observed in PRO suggests that, ultimately, professional experience in translation optimizes semantic processing for all word types. Further research is needed to clarify this issue.

**Form-related effects**. For BEG and PRO, BT was faster than FT with non-cognates, but not with cognates –as was the case for both groups in Experiment 1. Notice that this effect, together with a symmetrical performance for cognates, was found for HI in Experiment 1, as well as in previous studies with relatively proficient non-translators (de Groot et al., 1994, Experiment 1; Sánchez-Casas et al., 1992).

In the discussion of Experiment 1, we proposed that the presence of selective directionality effects for non-cognates in HI, but not in LOW, might be influenced by both L2 proficiency and translation expertise. Such an interpretation is applicable to the present contrast between BEG and PRO, which differed in both variables. However, ADV evidenced a different pattern, problematizing the explanation previously proposed. Further research is necessary to elucidate this point.

The only previous study on word translation controlling for both L2 proficiency and translation expertise (Christoffels et al., 2006) also found a cognate effect irrespective of both variables, but it did not report any directionality differences for either cognates or non-cognates in any group. The authors, however, do maintain that both L2 proficiency and translation expertise may influence cognate status effects on word-retrieval tasks.

Intriguingly, unlike Experiment 1, the analysis of the cognate status by concreteness interaction revealed all comparisons to be significant. This suggests that the relative contribution of the form- and meaning-based links between translation equivalents is influenced by the development of *formal* translation expertise. Finally, notice that BEG and PRO, while different in their overall RTs, had very similar concreteness and cognate-status effects, but this was not the case for ADV. Notice, also, that ADV differed from both BEG and PRO in terms of translation expertise, but not in terms of L2 proficiency. It might be surmised that massive modifications of connection strengths occur at both the form and the semantic levels during the early stages of, and due to, formal translation training, but that the system tends to approximate its initial patterns of connectivity –though with lower thresholds overall– after several years of translation practice (PRO).

#### Summary

As stated before, BEG and ADV, on the one hand, and ADV and PRO, on the other, differed in translation expertise but not in L2 proficiency. Thus, this second experiment offers further evidence that translation expertise may influence lexical processing independently of L2 proficiency. It also suggests that whereas the acquisition of formal translation skills does not modulate specific aspects of bilingual processing – specially those implicated in word reading –, it does have an impact on the relative strengthening of both form- and meaning-based links supporting word translation. The exact nature of this influence, however, remains to be elucidated.

## GENERAL DISCUSSION

This study examined the role of translation-specific skills in bilingual lexical retrieval processes. Specifically, we explored the impact of different types and levels of translation abilities on word reading and word translation. Our results showed that translation expertise, as a variable different from L2 proficiency, may play an important role in the observed effects.

In all groups, word reading was consistently faster than translation and gave no signs of being semantically mediated. No cognate effects were observed in this task, either. Also, reading was faster in L1 than in L2 for four of the five groups – only HI, in Experiment 1, had comparable RTs for these tasks. Hence, our first hypothesis, which predicted longer latencies for L2 reading irrespective of both type and level of translation expertise, was mostly, though not entirely, confirmed.

Our second hypothesis was that BT would be overall faster than FT only in non-translators. The groups possessing formal translation training did not show overall asymmetries in word translation, and neither did HI in Experiment 1. This suggests that both any level of formal translation expertise and a high level of informal translation expertise may eliminate translation asymmetries. However, our results do not allow us to rule out L2 proficiency as another factor contributing to this effect in non-translators.

Despite the above findings, selective directionality effects were found for different combinations of concreteness and cognate-status values, and these differed among the groups. It appears that not only the level but also the type of translation expertise results in specific modifications of the strengths of inter-equivalent links at the levels of meaning and form. Whereas the concreteness and the cognate-status effects shown by LOW and HI in Experiment 1 were mostly in agreement with the results of previous studies conducted with non-translators, the groups possessing formal translation skills deviated from the most recurrent findings in the literature.

We had also hypothesized that RTs in both reading and translation would significantly decrease as translation expertise – be it formal or informal – increased. Thus postulated, this hypothesis must be rejected, given that no such effects occurred in Experiment 1, and only one of the groups in Experiment 2 performed significantly slower than the others (BEG relative to ADV and PRO). It follows that the impact of the level and the type of translation expertise has a greater influence on the *relative strength* of the semantic and formal links involved in lexical processing than on the overall speed with which the tasks can be accomplished. This result, incidentally, suggests that the modifications induced by formal translation expertise in the lexical domain are more marked in the early than in the late stages of training and practice.

Importantly, our results further suggest that the role of L2 proficiency in translation-related effects may have been overemphasized in the literature. Previous studies have assumed a causal relationship between L2 competence and directionality effects, and vice versa. For example, in discussing the results of their word-translation study, Guasch et al. (2008, p. 289) affirm that “the influence of semantic relations depends on the participants’ level of [L2] proficiency.” For their own part, and reversing the line of causality, Christoffels et al. (2003, p. 206) claim that “[t]he equivalent RTs in both translation directions therefore suggests that our participants were relatively proficient in L2.” Our present findings suggest that, in addition to L2 proficiency, translation expertise may constitute another critical subject-variable underlying these effects.

In Experiment 1, differences between LOW and HI can be explained by both L2 proficiency or translation expertise. However, in Experiment 2, the differences observed between BEG and ADV and between ADV and PRO can only be explained in terms of translation expertise. Thus, our study indicates that some of the effects typically attributed to L2 proficiency differences may also be partially explainable as a function of translation expertise. This view is compatible with important theoretical positions and empirical findings in the literature.

First, there is a wide theoretical consensus that translation expertise, even in terms of strictly linguistic aspects, involves more than L2 competence (Obler, 1983; PACTE Group, 2000). Indeed, models of translation expertise recognize the translation-exclusive ‘transfer subcompetency’ as a skill that is distinct from L1 or L2 competence (PACTE Group, 2000). Second, evidence gleaned from case reports of brain-lesioned bilinguals suggests that the neural routes supporting translation are functionally independent from those supporting monolingual production in either language (Paradis, 1984, 1994). Moreover, the inhibition of certain brain areas via direct electrostimulation interferes with monolingual processing in L1 and L2 without impairing translation skills, warranting the conclusion that “the process of translation must use neurocognitive pathways spatially distinct from these sites which have been identified as involved in reading or naming” (Borius et al., 2012, p. 620) – for a review of further supporting evidence, see García (2013). This would imply that at least some of the connections implicated in translation are separate from those involved in monolingual tasks in L1 and L2, so that they could be independently strengthened or weakened – depending on how long the subject has been practicing translation, as opposed to monolingual processing. Third, previous studies comparing professional translators with non-translators, matched for L2 proficiency, showed that translation expertise can modulate linguistic (Ibáñez et al., 2010) and executive control (Yudes et al., 2011) processes independently of proficiency in the foreign language. Taken together, these data suggest that at least some inter-equivalent links are more sensitive to translation practice than L2 competence.

This conclusion has important implications for studies on bilingual lexical processing, in general, and word translation, in particular. The mainstream position that translation-related effects are modulated directly by L2 proficiency cannot be fully embraced until these are replicated in studies comparing low- and high-L2-proficiency groups matched for translation expertise. Also, our results indicate that future studies should not only control for translation expertise, but also specify whether it was acquired informally or through field-specific training. Indeed, the present results showed that formal expertise in translation may modulate form- and meaning-level effects in ways that informal translation expertise does not.

Finally, our study has implications for testing competing models of bilingual memory organization. In particular, some of our results are incompatible with specific tenets of the Revised Hierarchical Model (Kroll and Stewart, 1994; Kroll et al., 2010), notably the claim that only FT is semantically mediated. On the other hand, a model that acknowledges the relative contribution of different types of connections for different word types within and across languages, such as the Bilingual Interactive Activation + model (Dijkstra and van Heuven, 2002), is better equipped to account for the results presently reported. For a point-by-point comparison of both models, see Brysbaert and Duyck (2010).

Note that the above conclusions are based on comparisons between groups formed via self-assessment questionnaires. While objective measures may offer very relevant information to separate groups, the evidence indicates that self-report instruments are also appropriate. Specifically, in the field of bilingualism, several studies attest to the validity of self-report data to discriminate between groups as a function of language competence. For example, Langdon et al. (2005) found 100% agreement in language-dominance judgments between self-ratings of language competence and frequency of use, and color-form, color-animal, and color-object naming-time differences in both L1 and L2. Similarly, Marian et al. (2007) showed that global measures of self-reported proficiency were generally predictive of language ability. By the same token, Gollan et al. (2012) reported that self-ratings, proficiency interviews, and performance on a multilingual naming test were statistically similar in classifying bilinguals into language-dominance groups.

As regards translation/interpreting expertise, virtually all available studies on the topic have successfully assessed this variable using self-report questionnaires exclusively. For instance, Carl and Kay (2011) and Hvelplund (2011) assessed the impact of translation expertise on cognitive resource allocation during translation by comparing eye-tracking and key-logging activity between student and professional translators. In both cases, participants were assigned to either group by considering questionnaire data only (specifically, sex, education, years of experience in translation, possession of translation degree). Similarly, studies comparing interpreters vs. non-interpreters (Padilla et al., 2005) and interpreting students (Liu et al., 2004; Tzou et al., 2011) on working memory tasks determined their participants’ level of translation expertise based exclusively on questionnaire data. Moreover, the only previous study assessing the impact of translation expertise on word translation (Christoffels et al., 2006) relied solely on self-ratings to determine the language production and comprehension skills of their participants (non-translators, foreign-language teachers, professional interpreters). Unlike the instruments used in these studies, our questionnaires included critical items to gather quantitative data about the participants’ linguistic (e.g., L1 proficiency, L2 competence) and translation-specific (e.g., proficiency in BT, proficiency in FT) skills, in addition to other relevant information, such as years of experience in translation. Most of these variables have been overlooked in previous studies on translation expertise. Critically, our study seems to be the only one offering quantitative information (via Likert scales) about the participants’ translation skills in each direction.

## LIMITATIONS AND SUGGESTIONS FOR FURTHER RESEARCH

Some of the limitations of the present study imply suggestions for further research. First, our sample was small. However, the groups’ sample sizes were similar to those in other relevant studies (Liu et al., 2004; Christoffels et al., 2006; Duyck and Brysbaert, 2008; Ibáñez et al., 2010; Carl and Kay, 2011). Moreover, our screening protocol included well-defined inclusion and exclusion criteria, and several key variables were controlled when forming groups. Second, it would be important to conduct further experiments on word reading and translation with groups offering a clearer demarcation of their L2 proficiency and translation expertise. In this sense, it would be critical to develop finer measures of translation practice intensity, operationalized as daily hours of practice (like we proposed in our questionnaire) or other relevant variables. Third, it is possible that the translation-related effects may also be partly related to uncontrolled lexical variables in the stimuli sets, such as word familiarity (La Heij et al., 1996) and AoA of the L1 words (Bowers and Kennison, 2011). While it is practically impossible to construct long stimuli lists which control for these variables in addition to the ones considered in our study, small subsets of stimuli could offer valuable indications in this respect. Fourth, only nouns were used in this study; it would be interesting to examine how other word classes (e.g., adjectives, verbs) are processed in similar tasks and how they are affected by the variables of concreteness and cognate status. Also, this study used isolated words as stimuli. However, lexical access processes during translation can be modulated by the sentential context (van Hell, 2005), which raises the question of whether the reported findings would be replicated in a context-rich paradigm. Finally, it would be interesting to examine how excitatory and inhibitory mechanisms are affected by translation expertise in both word reading and word translation, and whether the observed effects hold irrespective of which modality the translators are experts in – e.g., (written) translation vs. (oral) interpreting.

## CONCLUSION

Our results suggest that both the type and the level of translation expertise play a role in modifying semantic and form-level connections in the bilingual lexicon. The impact of translation expertise seems to be greater in word translation than in word reading. This finding has important theoretical and methodological implications for the study of within- and between-language processes in bilinguals. Empirical findings typically explained as a function of differences in L2 proficiency may be partly or even fully caused by differences in the type and level of translation expertise. By contemplating and discriminating these variables in their sampling criteria, future studies on bilingual memory organization may clarify the relative contributions of translation expertise and L2 proficiency to the observed effects.

## AUTHOR CONTRIBUTIONS

The experiment was conceived and designed by Adolfo M. García and Agustín Ibáñez. Data were acquired by Adolfo M. García and Alexander L. Houck, analyzed by Álvaro Rivera-Rei, David Huepe, Sumeer Chadha, Agustín Ibáñez, and Adolfo M. García, and interpreted by Adolfo M. García, Álvaro Rivera-Rei, Agustín Ibáñez, Maëva Michon, Sumeer Chadha, Alexander L. Houck, and Carlos G. Lezama. The manuscript was drafted by Adolfo M. García, Álvaro Rivera-Rei, Agustín Ibáñez, Maëva Michon, Carlos G. Lezama, Sumeer Chadha, and Alexander Lee Houck.

## Conflict of Interest Statement

The authors declare that the research was conducted in the absence of any commercial or financial relationships that could be construed as a potential conflict of interest.

## Footnotes

1 Note that strictly linguistic (as opposed to executive) effects induced by translation expertise are likely independent from which translation modality has been more extensively practiced (written translation vs. oral interpreting; García, 2014; Ibáñez et al., 2010).

2 www.python.org

3 www.pygame.org

4 This last criterion does not imply that one possible translation of a given stimulus is more correct or desirable than another. The word *fury*, in decontextualized tasks as the ones presently described, could be translated as either *furia* or as *ira*. However, since *fury* has been entered as an abstract cognate in our stimuli blocks, only its rendition as *furia* was empirically relevant to the present study. In other words, we were concerned with studying specific instances of ‘descriptive equivalence’ rather than possible instances of ‘theoretical equivalence’ (Toury, 1980).

5 However, see Schwartz et al. (2007) for indications that the degree of consistency between graphemic and phonological representations may influence cross-language activations in word reading.

6 Again, this does not mean semantic processing plays absolutely no role in cognate translation. Indeed, Kroll and Stewart (1994) showed that the translation of cognate items may be influenced by the categorical interference effect.
