# Supplementary material for: Word reading and translation in bilinguals: the impact of formal and informal translation expertise
Source: Front Psychol. 2014 Nov 12;5:1302. doi: 10.3389/fpsyg.2014.01302 (PMC4228976; doi:10.3389/fpsyg.2014.01302)
Supplement: Supplementary file 1 [file Data_Sheet_1.DOC]

**Supplementary material**

**Appendix 1: Questionnaire used in Experiment 1**

**Appendix 2: Questionnaire used in Experiment 2**

**Appendix 3: Full stimuli lists used in both experiments**

| **Stimuli blocks in Spanish** | **Stimuli blocks in English** |
| --- | --- |
| **Key: (COG=cognate / NON/COG=non/cognate /**  **ABS=abstract / CON=concrete)** | |
| **SP1 Block** | **EN1 Block** |
| director (COG, CON)  historia (COG, ABS)  dios (NCOG, ABS)  pluma (NCOG, CON)  solución (COG, ABS)  tío (NCOG, CON)  vampiro (COG, CON)  cansancio (NCOG, ABS)  galaxia (COG, ABS)  periodismo (NCOG, ABS)  mesa (NCOG, CON)  animal (COG, CON)  castigo (NCOG, ABS)  dedo (NCOG, CON)  análisis (COG, ABS)  actor (COG, CON)  familia (COG, ABS)  diccionario (COG, CON)  maní (NCOG, CON)  grandeza (NCOG, ABS)  enciclopedia (COG, CON)  satisfacción (COG, ABS)  paraguas (NCOG, CON)  niñez (NCOG, ABS)  lápiz (NCOG, CON)  hambre (NCOG, ABS)  radio (COG, CON)  perfección (COG, ABS)  papel (COG, CON)  cuero (NCOG, CON)  comedia (COG, ABS)  pereza (NCOG, ABS)  dulzura (NCOG, ABS)  tentación (COG, ABS)  pollo (NCOG, CON)  doctor (COG, CON)  falacia (COG, ABS)  pala (NCOG, CON)  tigre (COG, CON)  creencia (NCOG, ABS)  revista (NCOG, CON)  secretaria (COG, CON)  amistad (NCOG, ABS)  crisis (COG, ABS)  ancestro (COG, CON)  ceguera (NCOG, ABS)  corporación (COG, ABS)  cena (NCOG, CON)  conexión (COG, ABS)  miedo (NCOG, ABS)  banana (COG, CON)  rey (NCOG, CON)  mejora (NCOG, ABS)  nación (COG, ABS)  hija (NCOG, CON)  tomate (COG, CON)  control (COG, ABS)  hospital (COG, CON)  torpeza (NCOG, ABS)  vela (NCOG, CON)  foto (COG, CON)  hermandad (NCOG, ABS)  señal (COG, ABS)  codo (NCOG, CON) | director (COG, CON)  history (COG, ABS)  god (NCOG, ABS)  feather (NCOG, CON)  solution (COG, ABS)  uncle (NCOG, CON)  vampire (COG, CON)  tiredness (NCOG, ABS)  galaxy (COG, ABS)  journalism (NCOG, ABS)  table (NCOG, CON)  animal (COG, CON)  punishment (NCOG, ABS)  finger (NCOG, CON)  analysis (COG, ABS)  actor (COG, CON)  family (COG, ABS)  dictionary (COG, CON)  peanut (NCOG, CON)  greatness (NCOG, ABS)  encyclopaedia (COG, CON)  satisfaction (COG, ABS)  umbrella (NCOG, CON)  childhood (NCOG, ABS)  pencil (NCOG, CON)  hunger (NCOG, ABS)  radio (COG, CON)  perfection (COG, ABS)  paper (COG, CON)  leather (NCOG, CON)  comedy (COG, ABS)  laziness (NCOG, ABS)  sweetness (NCOG, ABS)  temptation (COG, ABS)  chicken (NCOG, CON)  doctor (COG, CON)  fallacy (COG, ABS)  shovel (NCOG, CON)  tiger (COG, CON)  belief (NCOG, ABS)  magazine (NCOG, CON)  secretary (COG, CON)  friendship (NCOG, ABS)  crisis (COG, ABS)  ancestor (COG, CON)  blindness (NCOG, ABS)  corporation (COG, ABS)  dinner (NCOG, CON)  connection (COG, ABS)  fear (NCOG, ABS)  banana (COG, CON)  king (NCOG, CON)  improvement (NCOG, ABS)  nation (COG, ABS)  daughter (NCOG, CON)  tomato (COG, CON)  control (COG, ABS)  hospital (COG, CON)  clumsiness (NCOG, ABS)  candle (NCOG, CON)  photo (COG, CON)  brotherhood (NCOG, ABS)  signal (COG, ABS)  elbow (NCOG, CON) |
| **SP2 Block** | **EN2 Block** |
| soledad (NCOG, ABS)  imperio (COG, ABS)  hombro (NCOG, CON)  campeón (COG, CON)  furia (COG, ABS)  mar (NCOG, CON)  universidad (COG, CON)  queja (NCOG, ABS)  talco (NCOG, CON)  madre (COG, CON)  santidad (NCOG, ABS)  dimensión (COG, ABS)  acoso (NCOG, ABS)  desastre (COG, ABS)  padre (COG, CON)  fiesta (NCOG, CON)  peligro (NCOG, ABS)  total (COG, ABS)  collar (NCOG, CON)  hotel (COG, CON)  interés (COG, ABS)  sábado (NCOG, ABS)  primo (NCOG, CON)  menú (COG, CON)  pan (NCOG, CON)  cámara (COG, CON)  custodia (COG, ABS)  desarrollo (NCOG, ABS)  color (COG, ABS)  cajón (NCOG, CON)  tren (COG, CON)  fin (NCOG, ABS)  misterio (COG, ABS)  agencia (COG, CON)  cuerpo (NCOG, CON)  frialdad (NCOG, ABS)  criminal (COG, CON)  dueño (NCOG, CON)  viernes (NCOG, ABS)  razón (COG, ABS)  balde (NCOG, CON)  centro (COG, ABS)  moda (NCOG, ABS)  león (COG, CON)  posición (COG, ABS)  cable (COG, CON)  bombero (NCOG, CON)  umbral (NCOG, ABS)  ajo (NCOG, CON)  bondad (NCOG, ABS)  jungla (COG, CON)  pasión (COG, ABS)  fealdad (NCOG, ABS)  horno (NCOG, CON)  isla (COG, CON)  seguridad (COG, ABS)  pilar (COG, CON)  sensación (COG, ABS)  ducha (NCOG, CON)  prisa (NCOG, ABS)  gobierno (COG, ABS)  industria (COG, CON)  mujer (NCOG, CON)  ira (NCOG, ABS) | loneliness (NCOG, ABS)  empire (COG, ABS)  shoulder (NCOG, CON)  champion (COG, CON)  fury (COG, ABS)  sea (NCOG, CON)  university (COG, CON)  complaint (NCOG, ABS)  powder (NCOG, CON)  mother (COG, CON)  holiness (NCOG, ABS)  dimension (COG, ABS)  harassment (NCOG, ABS)  disaster (COG, ABS)  father (COG, CON)  party (NCOG, CON)  danger (NCOG, ABS)  total (COG, ABS)  necklace (NCOG, CON)  hotel (COG, CON)  interest (COG, ABS)  Saturday (NCOG, ABS)  cousin (NCOG, CON)  menu (COG, CON)  bread (NCOG, CON)  camera (COG, CON)  custody (COG, ABS)  development (NCOG, ABS)  color (COG, ABS)  drawer (NCOG, CON)  train (COG, CON)  end (NCOG, ABS)  mystery (COG, ABS)  agency (COG, CON)  body (NCOG, CON)  coldness (NCOG, ABS)  criminal (COG, CON)  owner (NCOG, CON)  Friday (NCOG, ABS)  reason (COG, ABS)  bucket (NCOG, CON)  center (COG, ABS)  fashion (NCOG, ABS)  lion (COG, CON)  position (COG, ABS)  cable (COG, CON)  fireman (NCOG, CON)  threshold (NCOG, ABS)  garlic (NCOG, CON)  goodness (NCOG, ABS)  jungle (COG, CON)  passion (COG, ABS)  ugliness (NCOG, ABS)  oven (NCOG, CON)  island (COG, CON)  security (COG, ABS)  pillar (COG, CON)  sensation (COG, ABS)  shower (NCOG, CON)  hurry (NCOG, ABS)  government (COG, ABS)  industry (COG, CON)  woman (NCOG, CON)  wrath (NCOG, ABS) |
| **SP3 (Reading Block)** | **EN3 (Reading Block)** |
| sierra (COG, CON)  lombriz (NCOG, CON)  deseo (COG, ABS)  casa (NCOG, CON)  tristeza (NCOG, ABS)  arpa (COG, CON)  presión (COG, ABS)  alma (NCOG, ABS)  envidia (COG, ABS)  máscara (COG, CON)  paz (NCOG, ABS)  baño (NCOG, CON)  piloto (COG, CON)  huevo (NCOG, CON)  anhelo (NCOG, ABS)  distancia (COG, ABS)  respeto (COG, ABS)  libro (NCOG, CON)  rata (COG, CON)  maldad (NCOG, ABS)  pared (NCOG, CON)  oquedad (NCOG, ABS)  raza (COG, ABS)  casino (COG, CON)  rodilla (NCOG, CON)  roca (COG, CON)  ansiedad (COG, ABS)  puja (NCOG, ABS)  nupcias (NCOG, ABS)  pasto (NCOG, CON)  división (COG, ABS)  museo (COG, CON)  cruce (COG, ABS)  torre (COG, CON)  chinche (NCOG, CON)  culpa (NCOG, ABS)  antena (COG, CON)  suma (COG, ABS)  torta (NCOG, CON)  lujuria (NCOG, ABS)  lluvia (COG, CON)  minuto (COG, ABS)  soberbia (NCOG, ABS)  puente (NCOG, CON)  águila (COG, CON)  olvido (NCOG, ABS)  puerta (NCOG, CON)  recesión (COG, ABS)  ciencia (COG, ABS)  pulpo (NCOG, CON)  siembra (NCOG, ABS)  saco (COG, CON)  desidia (NCOG, ABS)  ruiseñor (NCOG, CON)  succión (COG, ABS)  bota (COG, CON)  falta (COG, ABS)  cinta (NCOG, CON)  congoja (NCOG, ABS)  cebra (COG, CON)  algodón (NCOG, CON)  gasto (NCOG, ABS)  pingüino (COG, CON)  fusión (COG, ABS) | dinosaur (COG, CON)  execution (COG, ABS)  hammer (NCOG, CON)  welfare (NCOG, ABS)  astronaut (COG, CON)  proposal (COG, ABS)  warning (NCOG, ABS)  handle (NCOG, CON)  attitude (COG, ABS)  handbook (NCOG, CON)  libel (NCOG, ABS)  battery (COG, CON)  keyboard (NCOG, CON)  darkness (NCOG, ABS)  document (COG, CON)  obsession (COG, ABS)  likelihood (NCOG, ABS)  statue (COG, CON)  repetition (COG, ABS)  foot (NCOG, CON)  plastic (COG, CON)  difference (COG, ABS)  pineapple (NCOG, CON)  usefulness (NCOG, ABS)  kindness (NCOG, ABS)  raccoon (NCOG, CON)  fortune (COG, ABS)  ocean (COG, CON)  worry (NCOG, ABS)  liver (NCOG, CON)  computer (COG, CON)  justice (COG, ABS)  prowess (NCOG, CON)  language (COG, ABS)  chocolate (COG, CON)  awareness (NCOG, ABS)  burglar (NCOG, CON)  reaction (COG, ABS)  soldier (COG, CON)  concoction (NCOG, ABS)  flower (COG, CON)  kitchen (NCOG, CON)  fancy (NCOG, ABS)  conjunction (COG, ABS)  service (COG, ABS)  sandwich (COG, CON)  attachment (NCOG, ABS)  pillow (NCOG, CON)  machine (COG, CON)  trust (NCOG, ABS)  wrestler (NCOG, CON)  evidence (COG, ABS)  outline (NCOG, ABS)  backpack (NCOG, CON)  technique (COG, ABS)  office (COG, CON)  heaven (NCOG, ABS)  water (NCOG, CON)  mountain (COG, CON)  texture (COG, ABS)  death (NCOG, ABS)  camel (COG, CON)  competition (COG, ABS)  loudspeaker (NCOG, CON) |

**Appendix 4: Supplementary Data**

*Supplementary Data for Experiment 1*

Table 3. *Means, standard errors, 95% CI, mean RTs and SDs for main effect of level*

| Level | N | Mean | SE | -95.00% | +95.00% | Mean (ms) | SD (ms) |
| --- | --- | --- | --- | --- | --- | --- | --- |
| LOW | 11 | 2.81 | 0.02 | 2.76 | 2.85 | 772 | 110 |
| HI | 10 | 2.8 | 0.02 | 2.74 | 2.85 | 685 | 168 |

Table 4. *Means, standard errors, 95% CI, mean RTs and SDs for main effect of task*

| Task | N | Mean | SE | -95.00% | +95.00% | Mean (ms) | SD (ms) |
| --- | --- | --- | --- | --- | --- | --- | --- |
| L1R | 21 | 2.58 | 0.01 | 2.54 | 2.62 | 392 | 89 |
| L2R | 21 | 2.67 | 0.02 | 2.63 | 2.72 | 493 | 146 |
| BT | 21 | 2.91 | 0.02 | 2.86 | 2.95 | 852 | 212 |
| FT | 21 | 3.04 | 0.01 | 3 | 3.08 | 1183 | 348 |

Table 5. *Means, standard errors, 95% CI, mean RTs and SDs for main effect of cognate status*

| Cognate status | N | Mean | SE | -95.00% | +95.00% | Mean (ms) | SD (ms) |
| --- | --- | --- | --- | --- | --- | --- | --- |
| Cognate | 21 | 2.77 | 0.01 | 2.74 | 2.81 | 668 | 129 |
| Non-cognate | 21 | 2.83 | 0.01 | 2.79 | 2.86 | 792 | 164 |

Table 6. *Means, standard errors, 95% CI, mean RTs and SDs for main effect of concreteness*

| Concreteness | N | Mean | SE | -95.00% | +95.00% | Mean (ms) | SD (ms) |
| --- | --- | --- | --- | --- | --- | --- | --- |
| Abstract | 21 | 2.81 | 0.01 | 2.77 | 2.85 | 755 | 160 |
| Concrete | 21 | 2.79 | 0.01 | 2.76 | 2.82 | 706 | 132 |

Table 7. *ANOVA results for Experiment 1*

| Effect | *Df* | *F* | *p* | *Partial*  *eta-squared* |
| --- | --- | --- | --- | --- |
| Intercept | 1 | 26651,34 | .001** | 0.999 |
| LEVEL | 1 | 0.08 | .77 | 0.004 |
| Error | 19 |  |  |  |
| TASK | 3 | 239.12 | .001** | 0.926 |
| TASK*LEVEL | 3 | 23.86 | .001** | 0.557 |
| Error | 57 |  |  |  |
| COG | 1 | 90.78 | .001** | 0.827 |
| COG*LEVEL | 1 | 0.31 | .58 | 0.016 |
| Error | 19 |  |  |  |
| CONC | 1 | 20.30 | .001** | 0.516 |
| CONC*LEVEL | 1 | 0.02 | .89 | 0.001 |
| Error | 19 |  |  |  |
| TASK*COG | 3 | 52.88 | .001** | 0.736 |
| TASK*COG*LEVEL | 3 | 15.70 | .001** | 0.452 |
| Error | 57 |  |  |  |
| TASK*CONC | 3 | 4.82 | .004* | 0.202 |
| TASK*CONC*LEVEL | 3 | 1.97 | .12 | 0.094 |
| Error | 57 |  |  |  |
| COG*CONC | 1 | 10.67 | .004* | 0.360 |
| COG*CONC*LEVEL | 1 | 1.76 | .2 | 0.085 |
| Error | 19 |  |  |  |
| TASK*COG*CONC | 3 | 4.47 | .006* | 0.191 |
| TASK*COG*CONC*LEVEL | 3 | 1.45 | .23 | 0.071 |
| Error | 57 |  |  |  |
| *Note*: COG = cognate status; CONC = concreteness; significance levels: **p* < .01. ***p* < .001. | | | | |

*Supplementary Data for Experiment 2*

Table 8. *Means, standard errors, 95% CI, mean RTs and SDs for main effect of level*

| Level | N | Mean | SE | -95.00% | +95.00% | Mean (ms) | SD (ms) |
| --- | --- | --- | --- | --- | --- | --- | --- |
| BEG | 12 | 2.83 | 0.02 | 2.79 | 2.88 | 756 | 140 |
| ADV | 12 | 2.75 | 0.02 | 2.70 | 2.80 | 610 | 114 |
| PRO | 12 | 2.74 | 0.02 | 2.69 | 2.79 | 608 | 104 |

Table 9. *Means, standard errors, 95% CI, mean RTs and SDs for main effect of task*

| Task | N | Mean | SE | -95.00% | +95.00% | Mean (ms) | SD (ms) |
| --- | --- | --- | --- | --- | --- | --- | --- |
| L1R | 36 | 2.60 | 0.01 | 2.56 | 2.63 | 413 | 113 |
| L2R | 36 | 2.68 | 0.02 | 2.64 | 2.72 | 504 | 151 |
| BT | 36 | 2.90 | 0.01 | 2.87 | 2.92 | 824 | 167 |
| FT | 36 | 2.93 | 0.01 | 2.90 | 2.96 | 891 | 209 |

Table 10. *Means, standard errors, 95% CI, mean RTs and SDs for main effect of cognate status*

| Cognate status | N | Mean | SE | -95.00% | +95.00% | Mean (ms) | SD (ms) |
| --- | --- | --- | --- | --- | --- | --- | --- |
| Cognate | 36 | 2.75 | 0.01 | 2.72 | 2.77 | 600 | 129 |
| Non-cognate | 36 | 2.80 | 0.01 | 2.78 | 2.83 | 716 | 146 |

Table 11. *Means, standard errors, 95% CI, mean RTs and SDs for main effect of concreteness*

| Concreteness | N | Mean | SE | -95.00% | +95.00% | Mean (ms) | SD (ms) |
| --- | --- | --- | --- | --- | --- | --- | --- |
| Abstract | 36 | 2.78 | 0.01 | 2.75 | 2.81 | 676 | 144 |
| Concrete | 36 | 2.77 | 0.01 | 2.74 | 2.79 | 640 | 131 |

Table 12. *ANOVA results for Experiment 2*

| Effect | *Df* | *F* | *p* | *Partial*  *eta-squared* |
| --- | --- | --- | --- | --- |
| Intercept | 1 | 39975.49 | .001*** | 0.999 |
| LEVEL | 2 | 4.5 | .018* | 0.214 |
| Error | 33 |  |  |  |
| TASK | 3 | 218.08 | .001*** | 0.869 |
| TASK*LEVEL | 6 | 0.88 | .051 | 0.051 |
| Error | 99 |  |  |  |
| COG | 1 | 343.97 | .001*** | 0.912 |
| COG*LEVEL | 2 | 0.8 | .45 | 0.046 |
| Error | 33 |  |  |  |
| CONC | 1 | 40.40 | .001*** | 0.550 |
| CONC*LEVEL | 2 | 1.36 | .27 | 0.076 |
| Error | 33 |  |  |  |
| TASK*COG | 3 | 141.42 | .001*** | 0.811 |
| TASK*COG*LEVEL | 6 | 3.55 | .003** | 0.177 |
| Error | 99 |  |  |  |
| TASK*CONC | 3 | 9.37 | .001*** | 0.221 |
| TASK*CONC*LEVEL | 6 | 2.21 | .048* | 0.118 |
| Error | 99 |  |  |  |
| COG*CONC | 1 | 6.81 | .013* | 0.171 |
| COG*CONC*LEVEL | 2 | 0.59 | .55 | 0.035 |
| Error | 33 |  |  |  |
| TASK*COG*CONC | 3 | 3.46 | .019* | 0.095 |
| TASK*COG*CONC*LEVEL | 6 | 1.87 | .09 | 0.102 |
| Error | 99 |  |  |  |
| *Note*: COG = cognate status; CONC = concreteness; significance levels: **p* < .05. ***p* < .01. ***p* < .001. | | | | |

**Appendix 5: Sub-lists of English targets differing in their initial phoneme type**

Table 13. *Sub-list of English targets beginning with plosive phonemes*

| Stimuli | Length | Frequency |
| --- | --- | --- |
| dimension (COG, ABS) | 3 | 14965 |
| director (COG, CON) | 3 | 79813 |
| disaster (COG, ABS) | 3 | 14684 |
| galaxy (COG, ABS) | 3 | 11159 |
| garlic (NCOG, CON) | 2 | 11449 |
| paper (COG, CON) | 2 | 75383 |
| position (COG, ABS) | 3 | 77124 |
| powder (NCOG, CON) | 2 | 9639 |
| punishment (NCOG, ABS) | 3 | 9986 |
| tiredness (NCOG, ABS) | 3 | 264 |
| tomato (COG, CON) | 3 | 14864 |
| comedy (COG, ABS) | 3 | 9596 |
| MEANS | 2.75 | 27410.5 |

Table 14. *Sub-list of English targets beginning with fricative phonemes*

| Stimuli | Length | Frequency |
| --- | --- | --- |
| fallacy (COG, ABS) | 3 | 704 |
| Friday (NCOG, ABS) | 2 | 33827 |
| friendship (NCOG, ABS) | 2 | 10130 |
| harassment (NCOG, ABS) | 3 | 5882 |
| history (COG, ABS) | 3 | 114904 |
| hospital (COG, CON) | 3 | 58669 |
| photo (COG, CON) | 2 | 25492 |
| satisfaction (COG, ABS) | 4 | 12237 |
| Saturday (NCOG, ABS) | 3 | 32230 |
| signal (COG, ABS) | 2 | 16835 |
| solution (COG, ABS) | 3 | 32052 |
| vampire (COG, CON) | 3 | 2897 |
| MEANS | 2.75 | 28821.58 |

Table 15. *Sub-list of English targets beginning with vocalic phonemes*

| Stimuli | Length | Frequency |
| --- | --- | --- |
| actor (COG, CON) | 2 | 24848 |
| agency (COG, CON) | 3 | 56954 |
| analysis (COG, ABS) | 4 | 53840 |
| ancestor (COG, CON) | 3 | 6529 |
| animal (COG, CON) | 3 | 53127 |
| elbow (NCOG, CON) | 2 | 8536 |
| improvement (NCOG, ABS) | 3 | 18977 |
| industry (COG, CON) | 3 | 73055 |
| oven (NCOG, CON) | 2 | 11454 |
| owner (NCOG, CON) | 2 | 39492 |
| ugliness (NCOG, ABS) | 3 | 539 |
| umbrella (NCOG, CON) | 3 | 3900 |
| MEANS | 2.75 | 29270.92 |
